# A practical approach to febrile cancer patients:  Diagnostic stewardship in Oncology units

**DOI:** 10.12688/f1000research.154812.2

**Published:** 2024-10-16

**Authors:** Sridevi H.B., Anisha Maria Fernandes, Sanyo D'souza, Prashantha B., Pooja Rao, Suchitra Shenoy M

**Affiliations:** 1 Department of Pathology, Kasturba Medical College, Mangalore, Manipal Academy of Higher Education, Manipal, Karnataka, 576104, India; 2 Department of Microbiology, Kasturba Medical College, Mangalore, Manipal Academy of Higher Education, Manipal, Karnataka, 576104, India; 3 Department of Medical Oncology, Kasturba Medical College, Mangalore, Manipal Academy of Higher Education, Manipal, Karnataka, 576104, India; 4 Department of General Medicine, Kasturba Medical College, Mangalore, Manipal Academy of Higher Education, Manipal, Karnataka, 576104, India

**Keywords:** Oncology, Complete Blood Counts (CBC), Diagnostic stewardship, infections

## Abstract

**Introduction:**

Cancer and cytotoxic chemotherapy used for its treatment predispose to severe and often fatal infections. Prompt diagnosis and timely antibiotic therapy are crucial, with delays in therapy initiation having high mortality. Complete blood count (CBC) is an inexpensive, standardized, and preliminary investigation for the management and follow-up of cancer patients with diagnostic and prognostic value.

**Method:**

We studied the types of infections associated with various cancers treated with chemotherapy, their etiologies and susceptibility patterns, and the hematological profile of these patients as predictors of infection.

**Results:**

A total of 21 patients (12 solid and 9 hematological malignancies) presented with 31 febrile episodes. White Blood cell count (2079 cells/cu. mm), percentage of neutrophils (52.9%), absolute neutrophil (137.5 cells/cu. mm), and platelet count (1,77,507 cells/cu. mm) were significantly lower in the 11 patients with febrile neutropenia. The absolute lymphocyte count (412.7 cells/cu. mm) was reduced with a strikingly low Neutrophil-to-lymphocyte ratio (NLR) (6.07) in patients with neutropenia. Laboratory and radiological evidence were present in 14/15 episodes of hematological malignancies (p-0.218) whereas unexplained clinical sepsis was common in solid malignancies (p-0.0202). The majority of documented infections were bacterial, caused by gram-negative bacilli, often showing multi-drug resistance. Infectious etiologies were identified in 71.4% of the patients with febrile neutropenia for >5days. Bacterial infections developed within 2 days of neutropenia, whereas viral and fungal infections manifested in prolonged neutropenia. Multi-site infections and higher mortality rates were observed in patients with febrile neutropenia. (p<0.04)

**Conclusion:**

Febrile neutropenia is a common complication among patients receiving chemotherapy for cancer, with an increased risk of morbidity and mortality. Early, rapid, and accurate diagnosis is key to prompt intervention. Hematological parameters such as Total Leukocyte count, platelet count, NLR, and Platelet-to-lymphocyte ratio are promising biomarkers in conjunction with morphological changes in neutrophils, thus proving that CBC and peripheral smears are simple, easily available, cost-effective, and highly dependable screening tools, especially in resource-poor settings.

## Introduction

Better outcomes in cancer patients are seen with advancements in management using more aggressive and tailored regimens. Infections, however, continue to be a major cause of morbidity and mortality in cancer patients. ^1^ Immunosuppression due to malignancy as well as therapeutic modalities increase an individual’s susceptibility to infections. In hematological malignancies, the underlying immune deficiencies and myelosuppressive treatments are major contributing factors. ^2^ Obstruction, disruption of anatomical barriers and surgical interventions are additional risk factors for infections in solid tumours. ^3^ Apart from cytotoxic chemotherapy, radiotherapy and the use of medical devices such as catheter, stents and prosthesis carry an increased risk. ^3^

Common sites of infection are the blood stream, urinary tract, respiratory tract, and skin and soft tissue. Severe and persistent neutropenia, especially absolute neutrophil counts (ANC) <100 cells/ml, exponentially increases the risk of severe infections with bacterial, fungal, viral, and parasitic agents. ^2^^,^^3^ Bacterial infections are the most common and are often polymicrobial. ^2^^,^^3^ Studies from India, including one from our own center, show that infections are predominantly due to gram-negative bacilli. The increased incidence of multidrug-resistant organisms complicates its management. ^4^^–^^7^

A complete blood count (CBC) with peripheral smear examination is an important tool for monitoring patients undergoing chemotherapy. White blood cell (WBC) analysis is commonly used to investigate the risk of infection; however, in patients undergoing chemotherapy, it becomes challenging as the total WBC count is low and neutropenia may be observed. Infections stimulate the production of cytokines which in turn stimulates the production of immature granulocytes from marrow and this can be evidenced as ‘left shift’ in the smear examination. Toxicity changes in neutrophils in the form of toxic granules and vacuoles are observed in patients with infection. Absolute leukocyte counts, such as absolute neutrophil count, absolute monocyte count, neutrophil-to-lymphocyte ratio (NLR), platelet-to-lymphocyte ratio (PLR), platelet parameters such as plateletcrit (PCT), platelet distribution width (PDW), and mean platelet volume (MPV), have been extensively studied in predicting infections. ^8^^–^^10^

Increased mortality rates are observed when initial therapy with inappropriate antibiotics is initiated, even if the regimen is adjusted later based on microbiological investigations. ^11^ Early diagnosis with prompt and appropriate antibiotics ensures favorable outcomes. A robust diagnostic and antimicrobial stewardship program could guide oncologists and physicians in timely and effective empirical therapy.

Our study aimed to study the types of infections in patients with cancer undergoing chemotherapy, to identify the association of infections with specific cancers, the etiological agents, and their antimicrobial susceptibility profile, and to study the hematological profile of patients receiving chemotherapy during a febrile episode.

## Methods

In this prospective study, over a 1-year period, from August 2021 to July 2022, all cancer patients admitted to two of our tertiary care centers with signs and symptoms of infection were included. Patients on targeted therapies or immunomodulators, radiotherapy or post-stem cell transplant, and immediate post-surgical (<30 days postoperative) were excluded from the study.

The study was approved by the Institutional Ethics Committee of Kasturba Medical College, Mangalore (Reg No. ECR/541/Inst/KA/2014/RR-20) (DHR Reg. No. EC/NEW/INST/2020/742) on 22.07.2021 and assigned the protocol number IEC KMC MLR-07/2021/230. The committee permitted a waiver of consent to participate from the patients as patient details and data were sourced from the case files and there is no direct contact between the researcher and participant.

The patients were monitored for markers of infection with complete blood count (CBC) and C-reactive protein (CRP). Peripheral blood samples from the cases were studied using the Coulter ® DXH800 hematology analyzer (Beckman Coulter Inc., Miami, FL, USA). The CBC parameters included hemoglobin (Hb), total leukocyte count (TLC), platelet count (PLT), and differential leukocyte count (DLC). The neutrophil lymphocyte ratio (NLR) and platelet lymphocyte ratio (PLR) were calculated using the absolute neutrophil count, absolute lymphocyte count, and platelet count.

The peripheral smear stained with Leishman stain was used for morphological assessment of any evidence of infections, such as toxic changes, toxic granules, presence of immature granulocytes, presence of any virus-induced reactive lymphocytes, monocytosis, intracellular organisms, and/or hemoparasites. The immature granulocytes were calculated manually from a detailed peripheral smear examination.

Microbiological investigations were based on the clinical presentation, and an appropriate sample was collected. These investigations included microscopy, culture, serological tests, and molecular assays. Microscopy included Gram staining, Auramine-O staining, and KOH mounting. The culture was performed according to standard microbiological techniques for bacteria and fungi. Automated culture systems included the BacT/ALERT system (BioMerieux®, ^TM^ USA) for blood and sterile body fluids and MGIT (BD BACTEC® ^TM^) for suspected mycobacterial infections. Identification of the bacteria and yeasts, and antimicrobial susceptibility testing of the isolates were performed using the VITEK 2 Compact system (BioMerieux®, ^TM^ France), and minimum inhibitory concentration (MIC) was interpreted using the Clinical and Laboratory Standards Institute (CLSI) ^12^ guidelines. Serological and molecular tests were performed according to the clinician’s request.

Demographic and clinical data were extracted from the medical records, including the type of cancer, cancer treatment, clinical presentation, laboratory investigations, radiology findings, use of antibiotic/antifungal treatment, and outcome. Episodes of Febrile Neutropenia (FN) have been documented. FN was diagnosed in the presence of a single oral temperature of ≥38.3°C (101 °F) or 38.0°C (100.4 °F) for more than 1 h along with an absolute neutrophil count (ANC) ≤500/μl or ≤1000/μl with predicted rapid decline during the next 48hr. ^7^

The collected data were entered into MS Excel, analyzed, and presented in the form of tables, pie charts, and bar diagrams. The Mann-Whitney U test was used to assess the statistical differences between the neutropenic and non-neutropenic groups, and the chi-square test was used to analyze the distribution of categorical variables. All data were analyzed using JAMOVI software version 2.4.14. Statistical significance was set at p <0.05.

## Results

The study included 21 patients with solid or hematological malignancies, with a total of 31 febrile episodes. The mean age of the patients was 53.3 years with a male-to-female ratio of 3:1 (15 males and 6 females). There were 12 solid malignancies and 9 hematological neoplasms. Table 1 shows the various types of malignancies and distribution of cases presenting with febrile episodes.

**Table 1.** Various types of malignancies and distribution of cases presenting with febrile episodes.

Solid/Hematological Neoplasm	Final Diagnosis	Total Number of cases
**Solid malignancies**		**12 cases**
Esophagus	SCC	1
Testes	Mixed GCT with metastasis	1
Rectum	Adenocarcinoma with metastasis	1
Bone	Osteosarcoma	1
Breast	IDC	1
Lung	Adenocarcinoma-Metastatic	1
Lung	SCC	1
Breast	IDC	1
Ovary	Recurrent Serous ovarian carcinoma	1
Bone-Pelvis	Ewing’s sarcoma/PNET	1
Sigmoid colon	Adenocarcinoma	1
Ca colon	Adenocarcinoma	1
**Hematological malignancies**		**9 cases**
Thymus	T-LBL	1
Lymphoma	Follicular	1
CML Blast crisis	CML Blast crisis	1
HG B-NHL	HG-B-NHL	1
Pre-B ALL	Pre-B-ALL, BCR/ABL +	1
Lymphoma	DLBCL	1
Lymphoma	DLBCL	1
AML	AML	1
AML	AML	1

SCC: squamous cell carcinoma, GCT: germ cell tumor, IDC: invasive ductal carcinoma, PNET: Primitive neuroectodermal tumor, T-LBL: T-cell lymphoblastic lymphoma/leukemia, CML: Chronic myeloid leukemia, HG-B-NHL: High-grade B-cell lymphoma, ALL: Acute lymphocytic leukemia, Pre-B-ALL: Precursor B ALL, DLBCL: Diffuse large B-cell lymphoma, AML: Acute myeloid leukemia.

Febrile episodes in hematological malignancies were noted in older individuals compared to solid malignancies. There was a striking male predominance across all types of malignancy, presenting with febrile episodes. Table 2 shows the demographic and hematological profiles of the patients.

**Table 2.** Demographics and the hematological profiles of solid and hematological malignancy cases.

Parameters	All febrile episodes	Solid Malignancy	Hematological Malignancy	p value
Total no. of episodes	31 episodes	16 episodes	15 episodes	-
Age in years	54.5 (29-79)	48.4 (29-73)	60 (38-79)	0.11
Gender	3:1	2:1	3.5:1	0.612
FN episodes	11 episodes	6 episodes	5 episodes	0.809
Hemoglobin (gm/dL)	9.9 (6.6-13.5)	10.4 (8.1-12.9)	9.42 (6.6-13.5)	0.928
WBC count (cells/cu. mm)	8,034 (100-72,200)	5018.75 (100-19,200)	11,251.33 (100-72,200)	0.522
Neutrophil (%)	53.5 (1.4-94.4)	54.69 (4-94.4)	52.15 (2-88.6)	0.301
Lymphocyte (%)	30.0 (3.2-97.7)	32.63 (3.2-79.9)	27.22 (7-97.7)	0.323
Eosinophils (%)	1.7 (0-7)	2.02 (0-7)	1.42 (0-1.2)	0.097
Monocytes (%)	9.1 (0-25.5)	9.96 (0.8-25)	8.11 (0.1-25.5)	0.077
Basophils (%)	0.61 (0-4.2)	0.75 (0-4.2)	0.47 (0-0.9)	0.110
Left shift ( Figure 1)	11 cases	6/16 episodes	5/15 cases	0.810
Toxic granules ( Figure 1)	4 cases	2/16 episodes	2/15 cases	0.944
Absolute Neutrophil count (ANC) (cells/cu. mm)	4402.39 (12-18,124)	3877.25 (12-18,124)	4962.53 (14-15,162)	0.186
Absolute Leucocyte count (ALC) (cells/cu. mm)	909.44 (0-1905)	660.01 (96-1382.4)	1175.42 (73.8-5054)	0.151
Neutrophil lymphocyte ratio (NLR)	5.73 (0.01-29.49)	5.27 (0.11-29.49)	6.21 (0.01-26.84)	0.492
Platelet lymphocyte ratio (PLR)	307.23 (3.95-1121.24)	339.13 (106.7-1121.24)	273.20 (3.95-1025.07)	0.140
Platelets (cells/cu.mm)	1,67,490 (7000-55200)	1,80,875 (7000-552000)	15,3213.30 (20000-422000)	0.086
Microbiological evidence of infection	15	5	10	0.0244 *
Diagnosis based on radiological/other lab findings	9	5	4	0.389
Unexplained clinical sepsis	7	6	1	0.0202 *

* p <0.05, and statistically significant.

**Figure 1.**
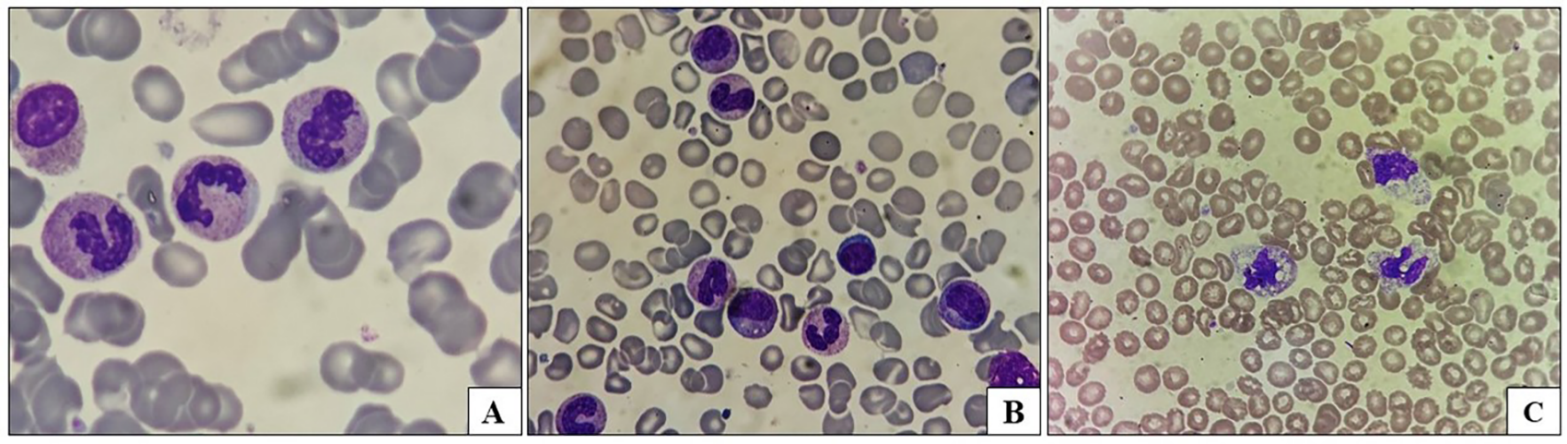
Peripheral smear findings: A: Toxic granules in neutrophils; B: Left shift; C: Toxic vacuolation in neutrophils.

There were a total of 11 febrile neutropenic episodes, of which 6 episodes were in solid malignancies [1 episode in IDC case, 1 in metastatic seminoma, 3 in bone tumors, and 1 in esophageal SCC], and 5 were in hematological malignancies [1 in LBL, 1 in ALL, 2 episodes in AML and one in DLBCL cases]. Table 3 shows the differences in the demographics and hematological profiles of febrile neutropenic and febrile non-neutropenic patients.

**Table 3.** Demographics and hematological profile of febrile neutropenic and febrile non-neutropenic patients.

Parameters	Febrile neutropenic episodes	Febrile non-neutropenic episodes	p value
Total no. of episodes	11 episodes	20 episodes	
Age in years	50.8 years	58.3 years	0.12602
Gender	3.5:1	2.25:1	0.472. Relative risk 6.83
Hemoglobin (gm/dL)	9.7	10.1	0.5892
WBC count (cells/cu. mm)	2079.1	11,310	0.00008*
Neutrophil (%)	21.3	71.1	< .00001*
Lymphocyte (%)	59.6	13.74	0.00026*
Eosinophils (%)	2.3	1.39	0.1074
Monocytes (%)	7.9	9.7	0.30302
Basophils (%)	0.7	0.5	0.90448
Left shift	6/11	5/20	0.099
ANC (cells/cu. mm)	137.5	6748.1	< .00001*
ALC (cells/cu. mm)	435.5	1170.1	< .00001*
NLR	0.6	8.6	< .00001*
PLR	399.5	256.5	0.98404
Platelets (cells/cu. mm)	81,181	2,14,960	0.0043*
Fatalities	3/11	1/20	0.0384*
Laboratory + Radiological evidence of infection	7	17	0.17346
Unexplained clinical fever	4	3	

WBC count, percentage of neutrophils, and absolute neutrophil and platelet counts were significantly lower in febrile neutropenic patients. Additionally, the absolute lymphocyte count was reduced, with a strikingly lower NLR ratio in patients with neutropenia than in those without neutropenia. However, there were no statistical differences in the CBC parameters of febrile episodes between solid and hematological malignancies.

Among the 31 febrile episodes during the study period, 16 episodes were observed in patients with solid organ tumors and 15 febrile episodes were observed in patients with hematological malignancies. Infectious etiology was documented with microbiological evidence in 15 episodes (48.4%) and supportive radiological or laboratory evidence in nine episodes (29.0%). Seven episodes (22.6%) were unexplained and were documented as clinical sepsis.

Microbiological evidence of infections was available in 10/15 febrile episodes in hematological malignancies compared to 5/16 febrile episodes in solid malignancies, which was statistically significant. Laboratory evidence, including radiological evidence, was present in 14/15 episodes in hematological malignancies compared to 10/16 episodes in solid malignancies, with a significant p-value of 0.0218. Unexplained clinical sepsis was statistically more common in solid malignancies (6/16 episodes) than in hematological malignancies (1/15).

Of the 18 bacterial infections, 15 showed documented microbiological evidence. Fungal and viral etiology was suspected in four episodes each, with microbiological confirmation of fungal etiology in two episodes (invasive aspergillosis) and viral etiology (SARS CoV-2) in one episode. Polymicrobial infections were noted in two episodes (6.5%).

Infectious etiologies were identified in 71.4% of patients with febrile neutropenia for > 5 days, whereas there was no confirmed laboratory diagnosis in 75% of patients with febrile neutropenia for ≤ 4 days. On average, bacterial infections were found to develop within 2 days following the development of neutropenia, whereas viral and fungal infections usually manifested if neutropenia persisted for 5 and >7 days, respectively.

Urinary tract infections accounted for 50% of the episodes, with respiratory tract infections (RTI), bloodstream infections (BSI), and skin and soft tissue infections (SSTI) in 37.5%, 20.8%, and 20.8% of the episodes, respectively. Multi-site infections were observed in four episodes (16.7%), of which three episodes were noted in patients with febrile neutropenia (p = 0.03). Urinary tract infections were the most common infections observed in patients with hematological malignancies (41.7%) and solid organ tumors (36.8%). Eighty % of BSI and 67% of RTI were observed in patients with hematological malignancies.

Most infections were caused by gram-negative bacilli (72.2%), with *E. coli* being the most common (69.2%). *Enterococcus faecalis* was observed in 80% of the infections caused by gram-positive cocci. All *E. coli* isolates were ESBL producers, and carbapenem resistance was observed in 100% of the *Acinetobacter baumannii* isolates and 50% of the *Klebsiella pneumoniae* isolates. All *E. faecalis* isolates were sensitive to beta-lactams, whereas all *Enterococcus faecium* isolates were vancomycin-resistant.

Mortality rates were significantly higher in patients with neutropenia than in those without neutropenia (p = 0.0384). Sixty-seven percent of fatalities among febrile neutropenia episodes had a prolonged neutropenia duration of > 12 days. Among fatalities, 75% were observed among patients with hematological malignancies, all of whom had bacterial infections with positive cultures.

## Discussion

Neutropenia is one of the most important risk factors for infection in cancer patients. The degree and duration of neutropenia corresponds to the severity of infection. Infection rates are inversely proportional to the absolute neutrophil count, with the highest incidence of infection seen with ANC <100μl. ^6^ Infectious etiologies are more likely to be identified in patients with longer durations of neutropenia (>7 days), ^3^^,^^13^ with a high risk of fungal infections. In our study, we did not observe a preponderance of infections during episodes of febrile neutropenia; however, multi-site infections were significantly more likely to occur. Infectious etiologies were identified in 75% of episodes with a longer duration of neutropenia. All cases of fungal pneumonia developed after prolonged neutropenia for more than a week and were caused by Aspergillus spp.

Bacterial infections are the most common type of infections in patients with cancer. ESKAPE ( *Enterococcus faecium, Staphylococcus aureus, Klebsiella pneumoniae, Acinetobacter baumannii, Pseudomonas aeruginos*a, and *Enterobacter* spp.) pathogens are prominent in bacterial infections and antibiotic resistance in cancer patients. ^14^ Gram negative bacilli accounted for 72.2% of infections, with similar rates reported in other studies. ^5^^,^^15^ In a study conducted by Bhat *et al.*, ^5^ *Pseudomonas aeruginosa* and *Klebsiella pneumoniae* were among the top gram-negative bacilli that cause infections. *E. coli* was the etiological agent in 50% of the infections we reported, responsible for 66.7% of UTI cases. The same study ^5^ reported only 14.7% infections due to *E. coli*, but similar rates (69.9%) of UTI.

Fungal infections are associated with indwelling devices and are often polymicrobial in nature. Prophylactic agents against *Candid*a spp. in patients with hematological malignancies have resulted in a surge in Aspergillus spp. infections. ^13^ As seen in our study, pneumonia in hematological malignancies is the most common presentation of aspergillosis.

The risk of viral infections increases with T cell suppression rather than neutropenia. Immunosuppression during intensive chemotherapy predisposes patients to RTI. Reactivation of HSV, VZV, HBV, CMV, and EBV has been reported. ^13^ Data published during the COVID-19 pandemic showed that cancer patients were more susceptible, with worse outcomes, including long COVID. ^16^ 75% of patients with viral infections presented with RTI. Although our study was performed during the pandemic, we surprisingly reported only one diagnosed case of SARS CoV-2, reflecting how the pandemic hindered patient follow-up. The patient developed a fatal secondary bacterial infection shortly thereafter. A study by Hemel *et al.,* ^16^ noted that cancer patients present with few symptoms of COVID-19 as immunosuppressants administered reduce the clinical severity, suggesting an alternative theory for the low rates of COVID infections in the study. We observed a single case of clinically diagnosed HSV.

A previous study at our center, ^5^ revealed that BSI was the most common infection (33.3%) in both hematological and solid malignancies. In contrast, our data showed that 50% of all enrolled patients presented with UTI, followed by RTI (37.5%). A study by Campos *et al.*, ^17^ reported a similar spectrum of infections in patients with solid malignancies. BSI is more common in Hematological malignancies, ^6^^,^^14^^,^^15^ as seen in 80% of our patients with bacteremia. A surge in polymicrobial infections has been reported in multiple studies, ^3^^,^^5^ however we report a low incidence of 6.7%.

The global increase in drug resistance among gram-negative organisms is alarming. ^5^^,^^6^^,^^13^^–^^15^ All the gram-negative isolates in our study were multidrug resistant, with 69.2% ESBLs and 23.1% carbapenem-resistant organisms (CROs). A study evaluating BSIs in malignancies ^15^ showed similar rates of resistance. They reported a higher mortality rate in drug-resistant gram-positive infections, which we did not observe in this study.

Unexplained fevers in cancer pose a challenge for clinicians. Hematological biomarkers have been increasingly used as both diagnostic and predictive factors in various medical conditions, especially in oncology and infectious conditions. Morphological changes in neutrophils in the form of changes in their shape, size, and internal complexity, with functional deviations in the form of deformability and disturbances in motility, have been detected in sepsis. ^18^ Complete blood count analysis with morphological assessment of leukocytes plays a pivotal role in diagnosing sepsis. Harada *et al.*, ^19^ in his study documented a band form percentage of >10% was correlated with an increased risk of bacteremia. Left shift of granulocytes with increased number of band forms, myelocytes, and metamyelocytes can be used as a screening modality, which additionally has prognostic significance in sepsis. ^20^ In the present study, left shift with the presence of immature granulocytes was noted more frequently in patients with solid malignancies than in those with hematological malignancies. However, the left shift was not statistically significant between the two groups or between febrile neutropenic and febrile non-neutropenic cases. In addition, we observed morphological changes in oncology patients with multiple febrile episodes. This re-emphasizes the importance of a simple ancient method of analyzing leukocyte morphology by peripheral smear examination in the early detection of sepsis. It can be a boon, especially in resource-limited centers where advanced diagnostic facilities are unavailable. ^21^ It is prudent to distinguish the toxic granules in the neutrophils due to infection from that of G-CSF in cancer patients. Subtle differences could help differentiate the two. The toxic granulation secondary to infection are dark coloured ranging from dark blue to dark black whereas those secondary to G-CSF are larger and purple coloured. The granulation caused by G-CSF is generally florid with large and more numerous granules having increased staining density that are noticed in almost all neutrophils as compared to infection induced granules. Pseudo-Pelger-Huet anomaly is an acquired neutrophil change noted in patient taking medications for transplant, cancer drugs etc. Presence of PPHA may pose diagnostic challenge in patients with history of cancer therapy and associated cytopenias to differentiate from myelodysplastic neoplasm associated Pelger-Huet anamoly. Careful peripheral smear examination for other features associated with MDS with clinical correlation is mandatory to differentiate. ^22^ Additionally, oncology patients receiving chemotherapy can have neutropenia, lymphopenia, and thrombocytopenia, which further compromises host immunity and hence increases the incidence of bacterial infections in such patients. ^23^

The neutrophil-to-lymphocyte and platelet-to-lymphocyte ratios have emerged as promising biomarkers in cancer-related particulars. ^24^^,^^25^ Zahorec *et al.* in 2001, were the first to propose neutrophil-to-lymphocyte ratio (NLR) as an important additional marker of infection in clinical practice which is directly proportional to disease severity. ^26^^,^^27^ More and more evidences are there proving NLR to be an independent predictor of poor survival in cancer patients. A study performed by Salciccioli JD *et al.* showed association of NLR with clinical progression of sepsis where NLR measure at the time of ICU admission was related to higher 28-day mortality and some studies have linked NLR and platelet-to-lymphocyte ratio (PLR) with short term or long-term mortality in sepsis. ^28^^–^^31^ Thus, proving these ratios to be a simple, cost effective and feasible biomarkers of sepsis in clinical practice. In solid malignancies, NLR and PLR parameters can be utilized in correlation with tumor size-node-metastasis (TNM) staging. The inflammatory cells and their mediators play a pivotal role in development and progression of neoplasms. Chemotherapy directly affects inflammatory cell and immune cells where recovery may be different from individual to individual and post-therapy changes affect the disseminated metastatic cell expression. Lymphocytes and neutrophil have an important role in tumor immune surveillance and can directly control the growth of cancer by cytotoxic activity and apoptosis by lymphocytes and suppression of cytolytic activity of immune cells by neutrophils. Neutrophils also secrete growth promoting factors in cancer. On the other side, platelets also secrete growth factors promoting proliferation and neoangiogenesis. ^32^^–^^34^ Thus, studying NLR and PLR can be of importance in assessing the poor prognosis in many cancers. ^35^^,^^36^ A meta-analysis covering 100 studies has stated that increased NLR is associated with adverse survival in numerous solid malignancies. ^37^ A lower NLR is an independent factor associated with high mortality and morbidity in sepsis. ^38^ Similar utility of NLR in predicting febrile neutropenia in cancer patients on chemotherapy and associated increased mortality and morbidity risk has been documented. ^39^^,^^40^ Variations of NLR values in accurately diagnosing bacteremia can be attributed to inflammatory response, marrow metastasis, infections, chemotherapy and associated hematological and lymphatic disorders. ^23^ A study by Hwang *et al.,* ^38^ in 2016 on 1395 patients, showed that initial NLR and subsequent persistent low or high values of NLR is a risk factor for predicting 28-day mortality with its increased diagnostic accuracy predicting sepsis in cancer patients. We observed a lower NLR in the patients with FN.

In the present study, PLR was higher in the febrile neutropenia group. Shen *et al.*, in a study of 5537 patients with sepsis, documented PLR as a prognostic biomarker for increased mortality. ^24^ Increased PLR is negatively associated with overall survival (OS) and can provide information on the therapeutic effectiveness of chemotherapy in solid malignancies. ^37^

In conclusion, febrile neutropenia is a common complication with a high risk of morbidity and mortality among patients undergoing chemotherapy for malignancies. Multiple factors determine the patient's outcome, including the microbiological spectrum. Clinicians must understand the immune defects associated with specific cancers and their chemotherapy to anticipate infections. Appropriate antibiotics, both empirical and regimens tailored to the microbiology report, greatly reduce morbidity and mortality, especially considering the growing concern of multidrug resistance. Diagnostic modalities continue to evolve but are limited with respect to the spectrum of infections, cost, and availability. Early and rapid diagnosis remains the key to prompt intervention, for which reliable biomarkers are needed. Hematological parameters, such as TLC, platelet count, NLR, and PLR, are promising tools. These parameters were integrated with peripheral smear examination to look for morphological changes notably in neutrophils, and the presence of immature granulocytes continues to be a simple, easily available, cost-effective, and highly dependable screening tool, especially in resource-poor settings.

### Ethics and consent

Approval for this study was obtained from the Institutional Ethics Committee (Kasturba Medical College, Mangalore), Reg No. ECR/541/Inst/KA/2014/RR-20, DHR Reg. No. EC/NEW/INST/2020/742. The approval was given on 22.07.2021 with protocol number IEC KMC MLR-07/2021/230. The committee permitted a waiver of consent to participate from the patients as patient details and data were sourced from the case files and there is no direct contact between the researcher and participant.

### Link for Peer review

https://datadryad.org/stash/share/mDwqq0ZbZTXjIysjMD04gccBEV_Eg6TYwS5LRcsTEwI

## Data Availability

Data excel sheet for “ A practical approach to febrile cancer patients: Diagnostic stewardship in Oncology units” https://doi.org/10.5061/dryad.7sqv9s51w ^41^ This project contains the following underlying data:
•
Dryad_Data_Collection.xlsx • README_file.txt.txt (The file includes the title with the authors who have made contributions to the study, duration, location, funding and sharing and access information) Data are available under the terms of the Creative Commons Zero “No rights reserved” data waiver (CC0 1.0 Public domain dedication).
