# Supplementary material for: A practical approach to febrile cancer patients:  Diagnostic stewardship in Oncology units
Source: F1000Res. 2024 Oct 16;13:997. Originally published 2024 Sep 2. [Version 2] doi: 10.12688/f1000research.154812.2 (PMC11621608; doi:10.12688/f1000research.154812.2)
Supplement: Peer review history

# Reviewer report: Reviewer response for version 2

**DOI:** 10.5256/f1000research.173195.r332383

**Published:** 2024-12-05

**Authors:** Dr Monali Gupta

**Affiliations:** 1 Clinical Fellow, Histopathology and Haematopathology, King's College Hospital NHS Foundation Trust, London, UK

The authors have included all the points in this revision that I had suggested earlier. The microscopic images of the peripheral smears are of good quality.

Is the work clearly and accurately presented and does it cite the current literature?

Partly

If applicable, is the statistical analysis and its interpretation appropriate?

Yes

Are all the source data underlying the results available to ensure full reproducibility?

Yes

Is the study design appropriate and is the work technically sound?

Yes

Are the conclusions drawn adequately supported by the results?

Yes

Are sufficient details of methods and analysis provided to allow replication by others?

Partly

Reviewer Expertise:

Hematopathology, flow cytometry, cell counters, leukaemia, lymphoma, myeloma, MDS/MPNs, cancer genomics, AI

I confirm that I have read this submission and believe that I have an appropriate level of expertise to confirm that it is of an acceptable scientific standard.

# Reviewer report: Reviewer response for version 2

**DOI:** 10.5256/f1000research.173195.r332382

**Published:** 2024-10-21

**Authors:** Jayashree Kulkarni

**Affiliations:** 1 Sri Shankara Cancer Hospital and Research Centre, Bangalore, India

I have gone through the revised version and found satisfactory implementation of my comments on the first version. Kindly correct the spelling of anomaly in the discussion section in the following sentence "Presence of PPHA may pose diagnostic challenge in patients with history of cancer therapy and associated cytopenias to differentiate from myelodysplastic neoplasm associated Pelger-Huet **anamoly** "

Is the work clearly and accurately presented and does it cite the current literature?

Yes

If applicable, is the statistical analysis and its interpretation appropriate?

Yes

Are all the source data underlying the results available to ensure full reproducibility?

Yes

Is the study design appropriate and is the work technically sound?

Yes

Are the conclusions drawn adequately supported by the results?

Yes

Are sufficient details of methods and analysis provided to allow replication by others?

Yes

Reviewer Expertise:

NA

I confirm that I have read this submission and believe that I have an appropriate level of expertise to confirm that it is of an acceptable scientific standard.

# Response

**Published:** 2024-10-21

**Authors:** Anisha Maria Fernandes

**Affiliations:** Microbiology, Manipal Academy of Higher Education, Manipal, Karnataka, India

Thank you for your comments. Will make the necessary changes.

# Reviewer report: Reviewer response for version 1

**DOI:** 10.5256/f1000research.169886.r322615

**Published:** 2024-10-01

**Authors:** Dr Monali Gupta

**Affiliations:** 1 Clinical Fellow, Histopathology and Haematopathology, King's College Hospital NHS Foundation Trust, London, UK

The article is well written in simple and clear words. It does say that it is a prospective study but it does not mention the time period of the study undertaken as the size of the study is small. It would be good to mention in the introduction.

 In the methods and analysis, the authors have mentioned that they have used DXH800 haematology analyser from Beckman Coulter. This analyser provides Immature granulocyte % fairly accurately. Have the authors taken the IG% from the analyser in the study or have they calculated it manually from the peripheral smears? Worth a mention in the article. 

 Reading and interpreting a peripheral smear is a skill and requires training. It is prudent to distinguish the toxic granules in the neutrophils due to infection or due to G-CSF in cancer patients. Has this been addressed? 

 I would also recommend to include some pictures of the peripheral smears showing left shift, toxic granulations, cytoplasmic vacuolations, reactive lymphocytes, aspergillosis. 

 Are neutrophil-to-lymphocyte and platelet-to-lymphocyte ratios validated sepsis parameters in a clinical setting? It would be worth clarifying about these with supporting literature as many of them are still research parameters? 

 I found this statement quite intriguing - "In solid malignancies, NLR and PLR parameters can be utilized in correlation with tumor size node-metastasis (TNM) staging." Can the authors throw light on this statement and cite the reference from where it is taken? 

 I think the article should be accepted for indexing with the above clarifications and recommendations.

Is the work clearly and accurately presented and does it cite the current literature?

Partly

If applicable, is the statistical analysis and its interpretation appropriate?

Yes

Are all the source data underlying the results available to ensure full reproducibility?

Yes

Is the study design appropriate and is the work technically sound?

Yes

Are the conclusions drawn adequately supported by the results?

Yes

Are sufficient details of methods and analysis provided to allow replication by others?

Partly

Reviewer Expertise:

Hematopathology, flow cytometry, cell counters, leukaemia, lymphoma, myeloma, MDS/MPNs, cancer genomics, AI

I confirm that I have read this submission and believe that I have an appropriate level of expertise to confirm that it is of an acceptable scientific standard, however I have significant reservations, as outlined above.

# Response

**Published:** 2024-10-08

**Authors:** Anisha Maria Fernandes

**Affiliations:** Microbiology, Manipal Academy of Higher Education, Manipal, Karnataka, India

Thank you for your valuable inputs.

 We have addressed your comments regarding:

1. Study period.
2. Method of calculation of immature granulocyte %.
3. Differentiation of toxic granules in the neutrophils due to infection or due to G-CSF.
4. Peripheral smears showing toxic granulations, left shift and toxic vacuolation in neutrophils.
5. Discussion and reference on utility of NLR and PLR as biomarkers of sepsis.
6. Clarification and reference for utilization of NLR and PLR parameters in correlation with tumor size node-metastasis (TNM) staging in solid malignancies.

# Reviewer report: Reviewer response for version 1

**DOI:** 10.5256/f1000research.169886.r322608

**Published:** 2024-09-23

**Authors:** Jayashree Kulkarni

**Affiliations:** 1 Sri Shankara Cancer Hospital and Research Centre, Bangalore, India

The authors have, in a very simple way created awareness of the importance of accurate diagnosis of infections using basic tools like CBC and its components (ANC, ALC, NLR and PLR). These are available in a most basic laboratory and can be implemented without much difficulty. 

 As far as peripheral smear examination is concerned, it may be difficult and labor intensive, especially in high volume cancer care centers.

 I suggest the authors include "other mimics of left shift should be ruled out like: 1. G-CSF (very commonly used in neutropenic patients) induced changes which also show course granulations and can be mistaken for toxic granules 2. Taxane induced Pseudo-Pelger Huet anomaly, which many may not be aware of.

 The language/grammar is very lucid.

 Adequate and appropriate references are provided.

 The article may be accepted for indexing with above mentioned suggestions.

Is the work clearly and accurately presented and does it cite the current literature?

Yes

If applicable, is the statistical analysis and its interpretation appropriate?

Yes

Are all the source data underlying the results available to ensure full reproducibility?

Yes

Is the study design appropriate and is the work technically sound?

Yes

Are the conclusions drawn adequately supported by the results?

Yes

Are sufficient details of methods and analysis provided to allow replication by others?

Yes

Reviewer Expertise:

Hematopathology

I confirm that I have read this submission and believe that I have an appropriate level of expertise to confirm that it is of an acceptable scientific standard.

# Response

**Published:** 2024-10-08

**Authors:** Anisha Maria Fernandes

**Affiliations:** Microbiology, Manipal Academy of Higher Education, Manipal, Karnataka, India

Thank you for your valuable inputs.

 We have addressed your comment regarding:

 Mimics of left shift such as G-CSF and Taxane induced Pseudo-Pelger Huet anomaly.
